# Affordability of Medical Care Among Medicare Enrollees

**DOI:** 10.1001/jamahealthforum.2021.4104

**Published:** 2021-12-10

**Authors:** Jeanne M. Madden, Susmitha Bayapureddy, Becky A. Briesacher, Fang Zhang, Dennis Ross-Degnan, Stephen B. Soumerai, Jerry H. Gurwitz, Alison A. Galbraith

**Affiliations:** 1 Department of Pharmacy and Health Systems Sciences, School of Pharmacy and Pharmaceutical Sciences, Bouvé College of Health Sciences, Northeastern University, Boston, Massachusetts; 2 Department of Population Medicine, Harvard Medical School, Harvard Pilgrim Health Care, Boston, Massachusetts; 3 Meyers Primary Care Institute, Worcester, Massachusetts; 4 Division of Geriatric Medicine, University of Massachusetts Medical School, Worcester

## Abstract

**Importance:**

Cost-sharing requirements can discourage patients from seeking care and impose financial hardship. The Medicare program serves many older and disabled individuals with multimorbidity and limited resources, but little has been known about the affordability of care in this population.

**Objective:**

To examine the affordability of medical care among Medicare enrollees, in terms of the prevalence of delaying medical care because of costs and having problems paying medical bills, and risk factors for these outcomes.

**Design, Setting, and Participants:**

Cross-sectional analyses conducted from November 1, 2019, to October 15, 2021, used logistic regression to compare the probability of outcomes by demographic and health characteristics. Data were obtained from the 2017 nationally representative Medicare Current Beneficiary Survey (response rate, 61.7%), with respondents representing 53 million community-dwelling Medicare enrollees.

**Main Outcomes and Measures:**

New questions about medical care affordability were included in the 2017 Medicare Current Beneficiary Survey: difficulty paying medical bills, ongoing medical debt, and contact by collection agencies. A companion survey question asked whether individuals had delayed seeking medical care because of worries about costs.

**Results:**

Respondents included 10 974 adults aged 65 years or older and 2197 aged 18 to 64 years; 54.2% of all respondents were women. The weighted proportions of Medicare enrollees with annual incomes below $25 000K were 30.7% in the older population and 67.4% in the younger group. Self-reported prevalence of delaying care because of cost was 8.3% (95% CI, 7.4%-9.1%) among enrollees aged 65 years or older, 25.2% (95% CI, 21.8%-28.6%) among enrollees younger than 65 years, and 10.9% (95% CI, 9.9%-11.9%) overall. Similarly, 7.4% (95% CI, 6.6%-8.2%) of older enrollees had problems paying medical bills, compared with 29.8% (95% CI, 25.6%-34.1%) among those younger than 65 years and 10.8% (95% CI, 9.8%-11.9%) overall. Regarding specific payment problems, 7.9% (95% CI, 7.0%-8.9%) of enrollees overall experienced ongoing medical debt, contact by a collection agency, or both. In adjusted analyses, older adults with incomes $15 000 to $25 000 per year had odds of delaying care more than twice as high as those with incomes greater than $50 000 (odds ratio, 2.47; 95% CI, 1.82-3.39), and their odds of problems paying medical bills were more than 3 times as high (odds ratio, 3.37; 95% CI, 2.81-5.21). Older adults with 4 to 10 chronic conditions were more than twice as likely to have problems paying medical bills as those with 0 or 1 condition.

**Conclusions and Relevance:**

The findings of this study suggest that unaffordability of medical care is common among Medicare enrollees, especially those with lower incomes, or worse health, or who qualify for Medicare based on disability. Policy reforms, such as caps on patient spending, are needed to reduce Medical cost burdens on the most vulnerable enrollees.

## Key Points

**Question:**

Is medical care affordable among people in the Medicare program?

**Findings:**

In a nationally representative survey of 13 171 Medicare enrollees that asked several questions about the affordability of their medical care, the overall reported prevalence of ever delaying care during 2017 owing to worries about cost was 11%; the prevalence of having problems paying medical bills was also 11%, and 16% of the respondents experienced 1 of these 2 concerns or both. Unaffordability of care was associated with lower incomes, worse health, and being younger than 65 years with long-term disability.

**Meaning:**

The findings of this study suggest that the burden of medical care cost-sharing leads to care avoidance and financial strains among the most vulnerable patients despite insurance coverage through Medicare; equitable access to care may require targeted reforms aimed at reducing cost burden.

## Teaser

This survey study examines the affordability of health care and prevalence of payment difficulties among Medicare enrollees.

## Introduction

Medical care in the US frequently requires substantial payments from patients, even if they have health insurance. Ample research has reported that patient cost-sharing is a barrier that prevents many US residents from getting needed care, threatening health goals. Cost-sharing also may result in major medical debt or deplete household resources to the point that people cannot afford other necessities of life.^1,2,3,4,5^ The Medicare program insures most US residents aged 65 years or older as well as other adults with long-term disabilities or end-stage kidney disease. Medicare enrollees contribute only a small proportion of the total cost of their medical care through premiums and cost-sharing.^6,7^ Nevertheless, given the relatively large illness burden and modest incomes in this population, even heavily subsidized care can be difficult to afford. Standard Medicare requires payment of an annual deductible ($183 in 2017) followed by 20% coinsurance for physician and outpatient services. The average total out-of-pocket health spending for a Medicare enrollee in 2016 was $5460, including cost-sharing and premiums—nearly half of the average income from Social Security.^8,9^

Patient-reported cost burden and underuse of prescription drugs because of costs in Medicare have been studied extensively^10,11,12^ but there has been little research on the affordability of general medical services in Medicare.^13,14^ The published research on cost barriers to medical care has burgeoned with studies of working-age adults affected by expansions of insurance coverage under the Affordable Care Act,^15,16,17^ but these studies generally exclude the Medicare population.

The Medicare Current Beneficiary Survey (MCBS), the premier US survey of the Medicare population, recently added questions asking whether enrollees have been unable to afford care. We analyzed these new data to produce what is, to our knowledge, the first large-scale, focused examination of unaffordability of medical services in Medicare and risk factors for unaffordability. Specifically, we investigated self-reported delays in care owing to costs and problems paying medical bills.

## Methods

### Setting and Data Sample

Study data are from the 2017 MCBS, administered by the US Centers for Medicare & Medicaid Services to support policy development. The MCBS panels include about 15 000 Medicare enrollees annually. Most respondents sit for in-person, computer-assisted interviews 3 times per year over 4 years, providing information on health status, health behaviors, and use of health services. The 2017 response rate of the survey was 61.7%, averaged over returning and newly sampled respondents.^18^ We restricted our main study population to 13 171 respondents who had continuous Medicare enrollment from January 2017 through the 2017 Fall interview; at least 1 recorded response in each of the survey segments used in our analyses, including Winter 2018 questions that asked about affordability experiences during 2017; and no 2017 residency in a long-term care facility. Applying MCBS survey weights, our sample represented 52 968 679 community-dwelling enrollees nationwide (of an estimated 59 286 140 ever enrolled in Medicare in 2017). This study was approved by the Harvard Pilgrim Health Care Institutional Review Board. The MCBS methods and our use and reporting of MCBS data conform to the American Association for Public Opinion Research (AAPOR) reporting guideline and Strengthening the Reporting of Observational Studies in Epidemiology (STROBE) reporting guideline.^19,20^

### Main Outcome Measures

Interviewers asked respondents, in the past year, “have you had problems paying or were unable to pay any medical bills?” Although the second part of this question implies more severe unaffordability than the first, we refer more simply to this measure as “problems paying medical bills.” Only respondents who answered yes to having problems paying were asked the 2 follow-up questions: “Because of problems paying medical bills [in the past year], have you been contacted by a collection agency?” and “Do you currently have any medical bills that are being paid off over time?” (medical debt). All 3 questions were introduced into the MCBS in 2017. We also examined responses to a previously existing MCBS question that has been seldom studied:^14^ “[in the past year], have you delayed seeking medical care because you were worried about the cost?”

### Covariate Measures

Covariate measures included socioeconomic and clinical predictors of health care unaffordability identified in prior research.^12,21,22,23,24^ Information on age, sex, US region, and rurality are derived from Centers for Medicare & Medicaid Services administrative sources. Race, ethnicity, educational attainment, and marital status were self-reported. Income level is based on self-reports that MCBS enhances with imputation. All health-related measures were developed from self-reports, ensuring consistent measurement across MCBS respondents enrolled in both traditional Medicare and Medicare Advantage plans, because the latter are private managed care plans with no claims data available. These health outcomes include functioning (measured as counts of limitations in ordinary and instrumental activities of daily living^25^), general health status,^26^ depression and anxiety symptoms (based on Patient Health Questionnaire-9 [depression] and the Generalized Anxiety Disorder-2 [anxiety] scores^27,28^), and chronic illness burden (a count of self-reported ever having been diagnosed with cardiac disease, hypertension, diabetes, cancer, stroke, arthritis, dementia, psychiatric disorder [including depression], neurological disorder [excluding stroke], and pulmonary illness [including asthma and chronic obstructive pulmonary disease]). We created mutually exclusive categories of supplemental insurance type hierarchically as follows: any Medicaid enrollment during 2017, any Medicare Advantage plan enrollment but no Medicaid, traditional fee-for-service (FFS) Medicare enrollment with employer-sponsored supplemental insurance, FFS with self-purchased Medigap plan or other supplement including US Veterans Administration benefits, and FFS with no supplement reported (Table 1) by combining information from self-reports and Centers for Medicare & Medicaid Services administrative records.

**Table 1.** Characteristics of Community-Dwelling Medicare Enrollees in 2017, by Major Age Category^a^

Characteristic	Age 18-64 y		Age ≥65 y	
	Percentage^b^	No.	Percentage^b^	No.
No. (weighted No.)	NA	2197 (8 172 132)	NA	10 974 (44 796 547)
Age, y				
18-54	43.0	1510	NA	NA
55-64	57.0	687	NA	NA
65-74	NA	NA	57.0	4217
75-84	NA	NA	31.1	4435
≥85	NA	NA	11.9	2322
Sex				
Female	47.4	1021	55.3	6124
Male	52.6	1176	44.7	4850
Race				
Black	18.3	471	8.4	885
White	70.1	1493	82.4	9202
Other^c^	8.9	178	6.8	624
Ethnicity				
Hispanic	9.4	219	7.7	940
Non-Hispanic	90.0	1965	91.8	9982
Educational level				
No high school diploma	20.7	460	14.5	1890
High school diploma	32.8	836	25.0	2909
Some college	35.7	717	30.0	3191
Bachelor’s degree or higher	10.1	169	30.2	2945
Annual income, $				
<15 000	45.3	1227	14.9	1847
15 000-25 000	22.1	434	15.8	1937
25 000-50 000	17.9	306	27.5	3111
>50 000	14.7	230	41.8	4079
Marital status				
Married	34.6	573	56.1	5585
Widowed	6.3	85	24.0	3431
Single, divorced, or separated	59.0	1537	19.8	1949
Region				
Northeast	19.2	381	18.4	1962
Midwest	20.5	508	22.1	2592
West	16.8	325	22.0	2237
South	43.5	982	37.4	4182
Urban density				
Metro	76.9	1543	80.5	8331
Micro	14.4	411	12.4	1654
Rural	8.6	243	7.1	989
General health status				
Excellent	4.1	134	19.1	2019
Very good	8.8	252	33.6	3606
Good	31.7	675	30.5	3426
Fair	33.8	718	12.8	1450
Poor	20.8	406	3.7	431
No. of chronic conditions				
0-1	19.7	602	26.4	2557
2-3	38.3	853	45.4	5018
4-10	40.7	712	27.3	3299
Functional limitations				
ADLs				
0	48.0	1200	75.2	7830
1-2	29.0	566	16.9	2110
3-6	23.0	431	7.8	1028
IADLs				
0	34.9	781	72.9	7675
1-2	39.2	831	20.5	2437
3-6	25.5	570	6.4	829
Depression (PHQ-9)				
None or minimal	33.6	709	75.1	2023
Mild to severe	55.2	1122	18.4	8063
Anxiety (GAD-2)				
No	56.9	1153	84.4	9101
Yes	32.0	678	9.0	985
Supplemental insurance^d^				
Medicaid	43.7	1188	10.2	1271
Medicare Advantage	18.3	318	30.1	3593
FFS with employer/retiree	13.7	209	26.9	2638
FFS with self-purchased or other	5.6	84	22.3	2457
FFS with no supplement	18.8	389	10.5	1015

Abbreviations: ADLs, activities of daily living; FFS, fee-for-service; GAD-2, Generalized Anxiety Disorder-2; IADLs, instrumental ADLs; NA, not applicable; PHQ-9, Patient Health Questionnaire-9.

^a^ Most enrollees qualify for Medicare based on age (≥65 years). Enrollees younger than 65 years qualify for Medicare based on long-term disability or end-stage kidney disease.

^b^ Weighted percentage results add to less than 100% owing to missing values. Information was missing on depression and anxiety symptoms for 7.3% and on race for 2.4%; all other variables <1% missing.

^c^ Other race included Asian, Native Hawaiian or Pacific Islander, American Indian or Alaska Native, and more than 1.

^d^ Supplemental insurance types were assigned hierarchically as follows: any Medicaid enrollment during 2017, any Medicare Advantage plan enrollment but no Medicaid, traditional fee-for-service (FFS) Medicare enrollment with employer-sponsored supplemental insurance, FFS with self-purchased Medigap plan or other supplement including US Veterans Administration benefits, and FFS with no supplement reported.

### Statistical Analysis

We estimated the proportions of community-dwelling Medicare enrollees within strata defined by our covariate measures. All data were analyzed separately for enrollees aged 18 to 64 years with disability and those aged 65 years or older. Next, we estimated the proportions responding yes to each affordability question, then the unadjusted prevalence of the 2 main affirmative outcomes (delaying care and problems paying) within the covariate strata. We identified differences where 95% CIs did not overlap among strata. We then used logistic regression analyses to evaluate the independent association of the covariates with the main outcomes while controlling for other factors. Supplemental insurance type may partly reflect self-selection based on unmeasured need for care or be associated with unmeasured social factors (eg, employment history, local conditions). We conducted sensitivity analyses without this covariate. We also ran extension analyses adding terms interacting insurance type with income level to test the hypothesis that the elevated risks attributable to lower incomes among non-poor (ie, income $15 000 or above) individuals might differ by insurance type (eg, Medicare Advantage could provide detectable protections). All study analyses were weighted^29^ to accurately represent the national population of community-dwelling Medicare enrollees and were conducted from November 1, 2019, to October 15, 2021, using SAS, version 9.3 (SAS Institute Inc).

## Results

### Characteristics of Medicare Enrollees

Of the 13 171 respondents, 10 974 were aged 65 years or older, 2197 were aged 18 to 64 years, and 54.2% of all respondents were female. Survey-weighted analyses found that community-dwelling Medicare enrollees aged 65 years or older were diverse in socioeconomic and health status, while enrollees aged 18 to 65 years were generally worse off on all socioeconomic and health measures (Table 1). For example, 30.7% of older adults vs 67.4% among those younger than 65 years had annual incomes lower than $25 000. The proportion of older adults with 4 or more chronic conditions was 27.3% vs 40.7% among those younger than 65 years. Nearly 4 of 5 (79.3%) older enrollees had some form of non-Medicaid supplemental coverage, while 43.7% of the younger enrollees had dual Medicaid coverage and 18.8% possessed no supplemental coverage

### Prevalence of Unaffordability Outcomes in Medicare

Among older enrollees, 8.3% (95% CI, 7.4%-9.1%) delayed care owing to cost and 7.4% (95% CI, 6.6%-8.2%) had problems paying medical bills (Table 2). Although these proportions were similar, the overlap between the 2 outcomes was limited: only 3.4% (95% CI, 2.9%-3.9%) experienced both forms of unaffordability. Accordingly, the proportion who had either form or both was substantial: 12.2% (95% CI, 11.1%-13.3%) of older enrollees. Among the population younger than 65 years, 25.2% (95% CI, 21.8%-28.6%) delayed care, 29.8% (95% CI, 25.6%-34.1%) had problems paying, and 38.0% (95% CI, 33.4%-42.7%) experienced either difficulty or both. For community-dwelling Medicare enrollees overall, these rates were 10.9% (95% CI, 9.9%-11.9%) for delayed care, 10.8% (95% CI, 9.8%-11.9%) for problems paying, and 16.2% (95% CI, 14.8%-17.6%) having either or both problems. Many enrollees with problems paying also had at least 1 of the 2 more-specific problems assessed in follow-up questions: among all enrollees aged 65 years or older, 5.0% (95% CI, 4.4%-5.6%) experienced difficulties with collection agency contact, paying bills over time, or both; this rate was 24.1% (95% CI, 19.9%-28.3%) among enrollees younger than 65 years and 7.9% (95% CI, 7.0%-8.9%) among enrollees overall.

**Table 2.** Prevalence of Delaying Medical Care Associated With Cost Worries and Problems Paying Medical Bills Among Medicare Enrollees in 2017, by Major Age Category and Overall

Affordability outcomes	Yes Response, % (95% CI)^a^		
	Age <65 y	Age ≥65 y	Overall
Delayed care due to cost worries	25.2 (21.8-28.6)	8.3 (7.4-9.1)	10.9 (9.9-11.9)
Problems paying/unable to pay medical bills (follow-up questions asked only if yes was indicated on problems paying)	29.8 (25.6-34.1)	7.4 (6.6-8.2)	10.8 (9.8-11.9)
Collection agency contact	18.5 (14.9-22.2)	3.3 (2.8-3.8)	5.6 (4.9-6.3)
Currently paying bills over time	15.0 (12.3-17.8)	3.6 (3.0-4.1)	5.3 (4.6-6.0)
Both collection agency contact and paying bills over time	9.5 (7.5-11.5)	1.8 (1.5-2.2)	3.0 (2.6-3.5)
Either collection agency contact or paying bills over time, or both	24.1 (19.9-28.3)	5.0 (4.4-5.6)	7.9 (7.0-8.9)
Both delayed care due to cost worries and problems paying/unable to pay	17.0 (14.1-19.9)	3.4 (2.9-3.9)	5.5 (4.9-6.2)
Either delayed care due to cost worries or problems paying/unable to pay, or both	38.0 (33.4-42.7)	12.2 (11.1-13.3)	16.2 (14.8-17.6)

^a^ Nonresponses were rare (0.1%-0.2%) and combined with negative responses to create dichotomous yes/no variables.

### Association of Unaffordability With Poor Health and Lower Income

Older adults who had poor general health status or multiple functional limitations experienced high rates of unaffordability of care (Figure, B), similar to the high rates we had observed for the population younger than 65 years as a whole. We also observed bivariate associations between multimorbidity and unaffordability among the older adults. Those in the 2 income bands below $25 000 were approximately twice as likely to experience unaffordability as their peers with incomes in the third highest band, $25 000 to $50 000, but the results were notably similar between the 2 lowest bands.

**Figure.**
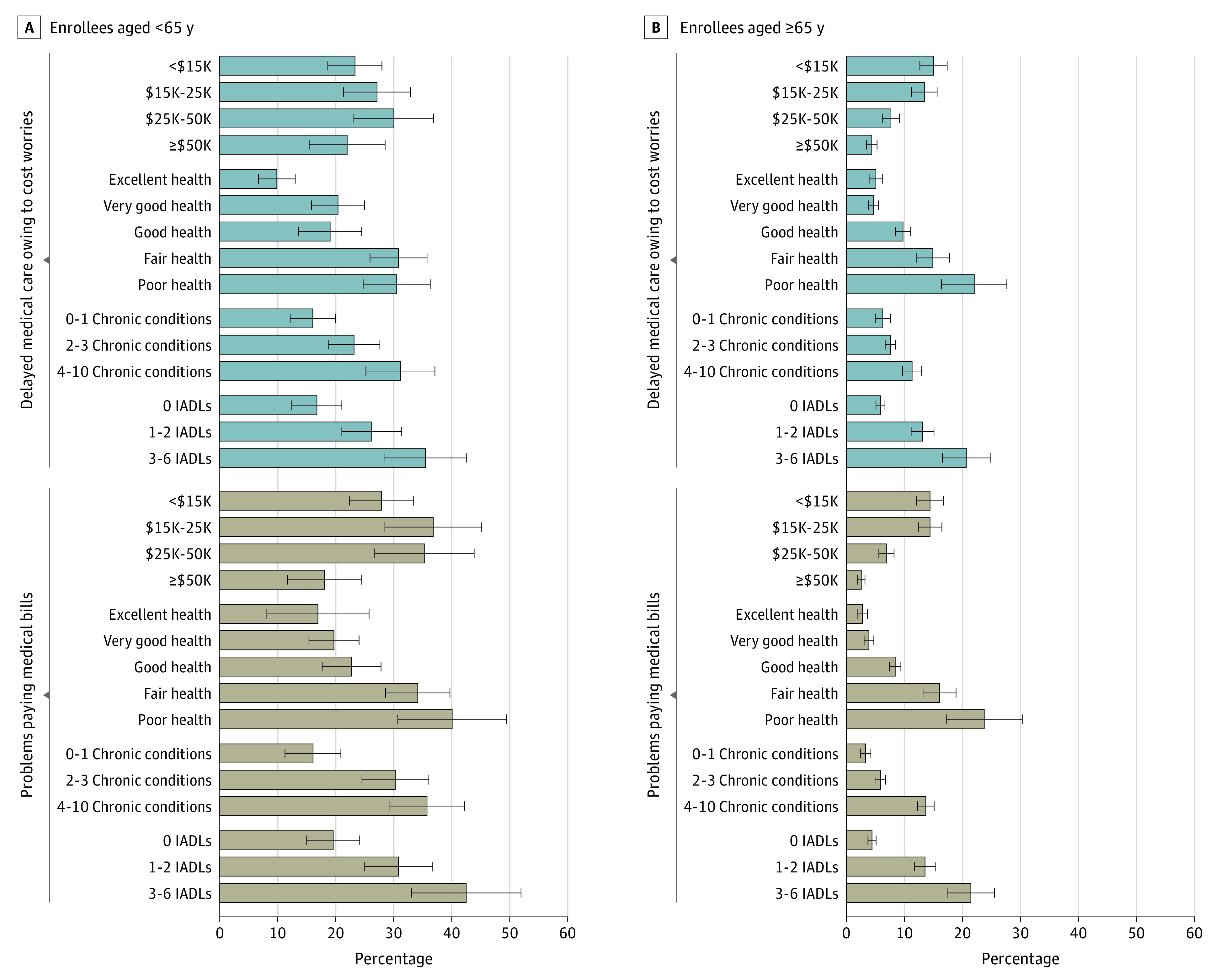
Variation in the Prevalence of Unaffordability of Care by Annual Income and Health Status for Medicare Enrollees Medicare enrollees aged 18 to 64 years (A) and 65 Years or older (B). IADLs indicates instrumental activities of daily living. Error bars represent 95% CIs around prevalence estimates.

The especially high rates of unaffordability across the individuals younger than 65 years are illustrated in the Figure, A. Because this population was relatively small, 95% CIs around these results were wide, but the associations between unaffordability and worse health, functioning, and illness burden were similar to those seen among the older population. Point estimates suggested more problems affording care among individuals with disabilities who had incomes in the 2 middle bands vs in the lowest and highest bands. Complete bivariate results are available in eTable 1 in the Supplement.

### Results From Logistic Regression Analyses

Multivariate model results for all enrollees are presented in Table 3. Among older individuals, the odds of both delayed care and problems paying bills were consistently and significantly higher in the least old group (aged 65-74 years), in all 3 lower income bands, and among those with 1 or more instrumental activities of daily living limitation or with depression or anxiety symptoms. The odds of experiencing problems paying medical bills were 3.4 times higher among near-poor enrollees (incomes $15 000-$25 000) than among those with incomes greater than $50 000 (odds ratio [OR], 3.37; 95% CI, 2.81-5.21), and the odds of delaying care because costs were 2.5 times higher (OR, 2.47; 95% CI, 1.82-3.39). Similar to our findings from the bivariate results, OR point estimates were highest among the near-poor group, who typically would not qualify for Medicaid.

**Table 3.** Adjusted OR Estimates of Delaying Care Owing to Costs and Having Problems Paying Medical Bills Among Medicare Enrollees, by Major Age Category

Characteristic	OR (95% CI)^a^			
	Age <65 y		Age ≥65 y	
	Delayed care owing to cost	Problems paying medical bills	Delayed care owing to cost	Problems paying medical bills
Respondents, No.	2064	2059	10 485	10 479
Age, y				
18-54	1 [Reference]	1 [Reference]	NA	NA
55-64	0.88 (0.64-1.22)	1.10 (0.80-1.51)	NA	NA
65-74	NA	NA	1 [Reference]	1 [Reference]
75-84	NA	NA	0.53 (0.45-0.63)^b^	0.61 (0.46-0.80)^b^
≥85	NA	NA	0.27 (0.19-0.37)^b^	0.33 (0.24-0.45)^b^
Sex				
Male	1 [Reference]	1 [Reference]	1 [Reference]	1 [Reference]
Female	1.25 (0.95-1.65)	1.07 (0.79-1.45)	1.14 (0.94-1.40)	1.00 (0.81-1.24)
Race				
White	1 [Reference]	1 [Reference]	1 [Reference]	1 [Reference]
Black	0.82 (0.53-1.29)	1.16 (0.81-1.68)	1.12 (0.82-1.53)	2.11 (1.55-2.88)^b^
Other^c^	0.69 (0.37-1.28)	0.74 (0.42-1.29)	1.34 (0.90-1.99)	1.08 (0.73-1.60)
Ethnicity				
Non-Hispanic	1 [Reference]	1 [Reference]	1 [Reference]	1 [Reference]
Hispanic	0.86 (0.43-1.70)	0.67 (0.38-1.20)	0.84 (0.62-1.15)	0.89 (0.59-1.35)
Education				
Bachelor’s degree or higher	1 [Reference]	1 [Reference]	1 [Reference]	1 [Reference]
No high school diploma	0.53 (0.32-0.88)^d^	0.64 (0.35-1.15)	0.93 (0.63-1.36)	1.35 (0.97-1.88)
High school diploma	0.71 (0.43-1.18)	0.73 (0.43-1.26)	0.92 (0.67-1.27)	1.12 (0.83-1.50)
Some college	0.73 (0.43-1.25)	0.86 (0.52-1.42)	1.03 (0.77-1.38)	1.23 (0.93-1.63)
Annual income, $				
≥50 000	1 [Reference]	1 [Reference]	1 [Reference]	1 [Reference]
<15 000	1.19 (0.65-2.18)	2.42 (1.08-5.42)^d^	2.39 (1.55-3.68)^b^	2.54 (1.65-3.89)^b^
15 000-25 000	1.22 (0.72-2.10)	2.86 (1.38-5.95)^e^	2.48 (1.82-3.39)^b^	3.37 (2.47-4.60)^b^
25 000-50 000	1.35 (0.76-2.41)	2.31 (1.13-4.72)^d^	1.50 (1.12-2.01)^e^	1.91 (1.32-2.74)^b^
Marital status				
Married	1 [Reference]	1 [Reference]	1 [Reference]	1 [Reference]
Widowed	0.75 (0.39-1.44)	0.83 (0.40-1.71)	0.99 (0.77-1.28)	1.12 (0.84-1.49)
Single, divorced, or separated	1.08 (0.78-1.50)	1.00 (0.72-1.38)	1.10 (0.85-1.41)	1.36 (1.07-1.73)^d^
Region				
Northeast	1 [Reference]	1 [Reference]	1 [Reference]	1 [Reference]
Midwest	1.09 (0.60-2.01)	1.44 (0.96-2.17)	1.18 (0.86-1.62)	1.31 (0.85-2.01)
West	1.40 (0.81-2.45)	1.31 (0.79-2.18)	1.27 (1.00-1.62)	1.08 (0.72-1.63)
South	1.25 (0.76-2.04)	1.82 (1.24-2.67)^e^	1.25 (1.00-1.56)	1.42 (1.00-2.01)
Urban density				
Metro	1 [Reference]	1 [Reference]	1 [Reference]	1 [Reference]
Micro	1.32 (0.90-1.95)	1.21 (0.83-1.75)	1.07 (0.83-1.39)	1.42 (1.09-1.85)^d^
Rural	1.52 (0.85-2.71)	0.95 (0.61-1.47)	0.82 (0.62-1.10)	0.99 (0.68-1.44)
General health status				
Excellent, very good, good	1 [Reference]	1 [Reference]	1 [Reference]	1 [Reference]
Fair or poor health	1.21 (0.86-1.70)	1.12 (0.77-1.62)	1.22 (0.94-1.59)	1.23 (0.96-1.57)
No. of chronic conditions				
0-1	1 [Reference]	1 [Reference]	1 [Reference]	1 [Reference]
2-3	1.13 (0.70-1.83)	1.87 (1.12-3.14)^d^	1.03 (0.79-1.35)	1.50 (1.11-2.04)^e^
4-10	1.35 (0.78-2.33)	1.77 (1.04-3.01)^d^	0.89 (0.67-1.18)	2.13 (1.59-2.85)^b^
Limitations in ADLs				
0	1 [Reference]	1 [Reference]	1 [Reference]	1 [Reference]
1-2	1.05 (0.70-1.58)	1.06 (0.76-1.47)	1.38 (1.06-1.80)^d^	1.05 (0.79-1.38)
3-6	1.18 (0.75-1.85)	1.31 (0.88-1.94)	1.43 (1.05-1.96)^d^	1.17 (0.83-1.65)
Limitations in IADLs				
0	1 [Reference]	1 [Reference]	1 [Reference]	1 [Reference]
1-2	1.26 (0.85-1.85)	1.30 (0.87-1.92)	1.68 (1.37-2.06)^b^	2.30 (1.73-3.05)^b^
3-6	1.70 (1.01-2.86)^d^	1.97 (1.28-3.05)^e^	2.21 (1.56-3.14)^b^	2.70 (1.77-4.12)^b^
Depression symptoms				
None or minimal	1 [Reference]	1 [Reference]	1 [Reference]	1 [Reference]
Mild to severe	1.54 (1.05-2.26)^d^	1.67 (1.16-2.42)^e^	1.93 (1.47-2.52)^b^	1.67 (1.23-2.27)^e^
Anxiety symptoms				
No	1 [Reference]	1 [Reference]	1 [Reference]	1 [Reference]
Yes	1.45 (1.06-1.98)^d^	1.85 (1.29-2.68)^e^	1.63 (1.18-2.26)^e^	2.02 (1.54-2.64)^b^
Supplemental insurance				
FFS with employer/retiree	1 [Reference]	1 [Reference]	1 [Reference]	1 [Reference]
Medicaid	1.28 (0.65-2.50)	1.01 (0.51-1.99)	1.65 (1.02-2.66)^d^	1.38 (0.80-2.37)
Medicare Advantage	2.32 (1.29-4.16)^e^	1.52 (0.78-2.94)	1.94 (1.40-2.70)^b^	2.01 (1.45-2.79)^b^
FFS with self-purchased or other	1.15 (0.42-3.13)	1.67 (0.67-4.15)	1.38 (0.95-2.01)	1.07 (0.71-1.62)
FFS with no supplement	1.71 (0.83-3.56)	1.55 (0.80-2.99)	2.42 (1.60-3.66)^b^	2.12 (1.39-3.23)^b^

Abbreviations: ADLs, activities of daily living; FFS, fee-for-service; IADLs, instrumental ADLs; NA, not applicable; OR, odds ratio.

^a^ Observations with missing values were dropped from the models, except for when missing on depression or anxiety.

^b^ *P* < .001.

^c^ Included Asian, Native Hawaiian or Pacific Islander, American Indian or Alaska Native, and more than one.

^d^ *P* < .05.

^e^ *P* < .01.

Several important factors were significantly associated with problems paying medical bills among older enrollees but not with delaying care because of cost; these factors included Black vs White race, being single vs married, and having 2 or more chronic conditions vs none.

Older enrollees in Medicare Advantage and those with traditional FFS Medicare enrollment with no supplemental coverage were more likely than those with FFS plus employer supplemental coverage to experience unaffordability, as defined by either of our 2 main outcome measures. Higher odds among older individuals with Medicaid were statistically significant in the case of delayed care, but not for problems paying (Table 3). Removal of the supplemental insurance variables from our models did not substantially affect the results, although the OR point estimates for all of the lower income bands were higher when the supplemental insurance variables were not present (eTable 3 in the Supplement). Extension analyses with interaction terms did not detect significant differences between Medicare Advantage and any other insurance category in the risks associated with lower incomes (eFigure and eTable 4 in the Supplement).

The population younger than 65 years was smaller and more uniformly disadvantaged; multivariate analyses yielded fewer statistically significant predictors (Table 3). Having 3 or more instrumental activities of daily living limitations vs none predicted both delayed care (OR, 1.70; 95% CI, 1.01-2.86) and problems paying (OR, 1.97; 95% CI, 1.28-3.05) in this population. Depression symptoms were also significantly associated with both delayed care (OR 1.54; 95% CI, 1.05-2.26) and problems paying (OR, 1.67; 95% CI, 1.16-2.42), as were anxiety symptoms, independently (OR 1.45; 95% CI, 1.06-1.98) for delayed care and problems paying (OR, 1.85; 95% CI, 1.29-2.68). Being in the 3 lower income categories, having 2 or more chronic conditions, and residing in the South were all statistically significant risk factors for problems paying medical bills, but not for cost-related delays in care.

## Discussion

Using newly available national survey questions about problems paying medical bills, we noted that 11% of all community-dwelling Medicare enrollees experienced such problems in 2017: more than 7% of adults older than 65 years and 30% of enrollees aged 18 to 64 years with disability. About 3 of 4 enrollees with problems paying medical bills had been contacted recently by a collection agency, had ongoing medical debt, or both. Separately, we estimated that 11% of enrollees delayed medical care in 2017 because they were worried about costs (8% of enrollees aged ≥65 years, 25% of those <65 years).

Although both the delayed care and problems with paying measures suggest that care was unaffordable, their meaning is distinct and the individuals affected were sometimes different: overall, 16% of enrollees had delayed care, problems paying, or both (12.2% of respondents aged ≥65 years, 38.0% of those <65 years). The distinctions point to the fundamental trade-offs patients face when care is too expensive: some may avoid care, potentially threatening their health, and others decide they must receive care and then have difficulty paying the bills. Having more chronic conditions was not associated with delaying care but was associated with having medical bills that could not be paid; very sick patients, in particular, may perceive themselves to have no choice but to receive medical care they cannot afford. However, functional deficits and symptoms of poor mental health were associated with both forms of unaffordability.

Of the 2 measures, only problems paying medical bills was significantly associated with 2 other known risk factors for financial hardship, Black race, and unmarried status,^3,30,31,32^ and was more than 3 times as likely to be found among near-poor individuals vs high income individuals. It may be that problems paying medical bills more directly measures financial difficulty, whereas, the question about delayed care owing to cost worries, although potentially more relevant to clinical care, has wording that could also wrap in emotional concerns or uncertainty about cost, complicating its interpretability. The problems paying medical bills question was accompanied by 2 follow-up questions that revealed most people with problems paying also had recent collection agency contact or were paying off bills over time; these more-specific responses provided helpful supporting evidence that the problems people reported were serious.^33^ The MCBS should consider adding questions that ask more directly about cost-related underuse of services, such as, “did not get a recommended procedure because it cost too much,” to better understand patient experiences of access.

We noted that, although lower incomes posed a higher risk of unaffordability, the risk appeared highest among older adults in the near-poor income band ($15 000-$25 000 annually), who are less likely to qualify for Medicaid assistance. The poorest individuals (<$15 000 annually), by contrast, may have had partial protection through Medicaid assistance. In a similar study,^12^ a relatively low risk of cost-related medication nonadherence was observed in this poorest stratum, who qualify for assistance from the Medicare Part D drug benefit’s low-income subsidy. Such results emphasize the power of insurance design policy to address affordability problems. Supplemental insurance type as a study covariate, however, is complicated because insurance type affects how much cost-sharing a patient faces but is also determined by a patient’s underlying socioeconomic characteristics, such as employment history. In the present study, dual Medicaid enrollment was associated with elevated risk, but not consistently, which is perhaps unsurprising given that people with Medicaid have lower cost-sharing but also very low incomes; these characteristics push in opposite directions. Likewise, Medicare Advantage is a relatively affordable option that disproportionately attracts enrollees with lower incomes.^34^ eTable 2 in the Supplement compares population characteristics among insurance types. More-detailed future study of the role of supplemental insurance in Medicare affordability is merited. Meanwhile, our results for the different supplemental insurance types may be helpful in identifying the patients at greatest risk.

Medicare is a nearly universal program among US residents aged 65 years or older, including individuals across the economic spectrum. Our data suggest that high rates of unaffordability are found among vulnerable segments of the older adult population, such as people with low incomes and those with high health care needs. Our findings also suggest that unaffordability is widespread among Medicare enrollees younger than 65 years, because low incomes and high illness burdens are typical in that population. We provide evidence that universal does not mean affordable or equitable.

The recent focus in US health care on the social determinants of health^35,36^ evinces the growing recognition that these factors—including unaffordable cost-sharing—can undo the efforts of programs and professionals to improve health. Clinicians and health systems can help meet health goals by asking patients directly about financial barriers and strategizing with them to identify more-affordable care options and access assistance programs. Health care professionals will first need awareness that many insured patients are nevertheless at risk for skimping on care or other hardships at home because of medical cost burdens.

Clinicians and policy makers can also press for public program changes to improve affordability for vulnerable patients. For example, cost burdens among lower income groups could be eased through expanded eligibility for Medicaid-administered Medicare Savings Programs, which assist with premiums and cost-sharing. Advocates for Medicare enrollees have also long sought the implementation of a cap on annual out-of-pocket spending in traditional FFS Medicare,^8^ which would be especially helpful to individuals who have high medical needs and could be set lower for enrollees with lower incomes. Caps on patient spending are standard in Medicare Advantage and non-Medicare commercial plans.

Other policy reforms could broaden access to Medigap supplemental coverage, which assists with patient costs. Medicare enrollees have only rare opportunities to sign up for a Medigap plan in most states, and Medigap is unavailable or unaffordable for many individuals younger than 65 years because of a lack of consumer protections for pre-existing conditions. Some states provide these needed protections, but federal reforms would reduce geographic disparities and put Medigap within reach for many more people.^37^

### Limitations

Our study has limitations. The findings rely on self-reported data. Respondents may have imperfect recall of experiences, such as their need for care, delays in care, and financial problems during the previous year.^38^ They could also underreport missed care or financial problems out of a sense of shame.^39^ At the same time, surveys offer a crucial window into phenomena that are poorly if ever captured in health system data. The MCBS is an exemplar of high-quality scientific methods, has undergone decades of validation and refinement, and brings forth perspectives representing millions of Medicare enrollees.

## Conclusions

Unaffordability is a serious problem for millions of patients enrolled in Medicare, resulting in delayed care and financial strains, such as debt and involvement with collection agencies. These new prevalence data may raise awareness, spur well-targeted efforts to alleviate hardships, and provide a foundation for other research that will bring additional insights and evaluate future Medicare reforms.

## Notes

Supplement. **eTable 1.** Detailed Prevalence Analyses of Delaying Care Due to Cost and Having Problems Paying Medical Bills, by Demographic and Health Characteristics **eTable 2.** Demographic and Health Characteristics of the Study Population by Supplemental Insurance Type **eTable 3.** Sensitivity Analysis—Multivariate Modeling Results Without Supplemental Insurance Type **eFigure.** Extension Analysis Examining Association Between Lower Income Status and Unaffordability by Supplemental Insurance Type: Estimated Odds Ratios Comparing Risk of Unaffordability Associated With Lower Incomes (as Indicated) Compared With Income $50K+, by Coverage Type **eTable 4.** Extension Analysis Examining Association Between Lower Income Status and Unaffordability by Supplemental Insurance Type: Multivariate Modeling Results With Interaction of Income Level and Supplemental Insurance Type, Varying the Insurance Reference Category
