# Supplementary material for: Affordability of Medical Care Among Medicare Enrollees
Source: JAMA Health Forum. 2021 Dec 10;2(12):e214104. doi: 10.1001/jamahealthforum.2021.4104 (PMC8796945; doi:10.1001/jamahealthforum.2021.4104)
Supplement: Supplement. — eTable 1. Detailed Prevalence Analyses of Delaying Care Due to Cost and Having Problems Paying Medical Bills, by Demographic and Health Characteristics eTable 2. Demographic and Health Characteristics of the Study Population by Supplemental Insurance Type eTable 3. Sensitivity Analysis—Multivariate Modeling Results Without Supplemental Insurance Type eFigure. Extension Analysis Examining Association Between Lower Income Status and Unaffordability by Supplemental Insurance Type: Estimated Odds Ratios Comparing Risk of Unaffordability Associated With Lower Incomes (as Indicated) Compared With Income $50K+, by Coverage Type eTable 4. Extension Analysis Examining Association Between Lower Income Status and Unaffordability by Supplemental Insurance Type: Multivariate Modeling Results With Interaction of Income Level and Supplemental Insurance Type, Varying the Insurance Reference Category [file jamahealthforum-e214104-s001.pdf]

Supplementary Online Content

Madden JM, Bayapureddy S, Briesacher BA, et al. Affordability of medical care among Medicare enrollees. *JAMA Health Forum*. 2021;2(12):e214104. doi:10.1001/jamahealthforum.2021.4104

eTable 1. Detailed Prevalence Analyses of Delaying Care Due to Cost and Having Problems Paying Medical Bills, by Demographic and Health Characteristics

eTable 2. Demographic and Health Characteristics of the Study Population by Supplemental Insurance Type

eTable 3. Sensitivity Analysis—Multivariate Modeling Results Without Supplemental Insurance Type

eFigure. Extension Analysis Examining Association Between Lower Income Status and Unaffordability by Supplemental Insurance Type: Estimated Odds Ratios Comparing Risk of Unaffordability Associated With Lower Incomes (as Indicated) Compared With Income $50K+, by Coverage Type

eTable 4. Extension Analysis Examining Association Between Lower Income Status and Unaffordability by Supplemental Insurance Type: Multivariate Modeling Results With Interaction of Income Level and Supplemental Insurance Type, Varying the Insurance Reference Category

This supplementary material has been provided by the authors to give readers additional information about their work.

© 2021 Madden JM et al. *JAMA Health Forum.*

Characteristic		Enrollees under 65 y		Enrollees 65 y and over	
		Delayed Care	Problems Paying	Delayed Care	Problems Paying
		% YES (95% C.I)	% YES (95% C.I)	% YES (95% C.I)	% YES (95% C.I)
**Age group**	18-54 y	26.0	28.8		
	55-64 y	24.6	30.6		
	65-74 y			10.1 (8.9-11.3)	8.6 (7.4-9.7)
	75-84 y			6.6 (5.6-7.5)	6.5 (5.4-7.5)
	85 y +			3.8 (3.1-4.5)	4.1 (3.2-5.0)
**Sex**	Female	29.0 (24.2-33.9)	33.7 (27.2-40.1)	9.5 (8.4-10.6)	8.5 (7.5-9.6)
	Male	21.8 (18.1-25.5)	26.4 (22.5-30.2)	6.8 (5.8-7.7)	5.9 (5.2-6.7)
**Race**	Afro Am	22.0 (15.5-28.4)	31.2 (23.5-38.9)	13.1 (9.3-16.8)	18.6 (14.2-22.9)
	White	26.7 (22.0-31.3)	29.8 (24.8-34.9)	7.3 (6.5-8.1)	5.9 (5.1-6.7)
	Other	22.3 (15.3-29.2)	25.9 (18.5-33.4)	12.0 (8.2-15.7)	8.7 (6.3-11.0)
**Ethnicity**	Hispanic	20.3 (12.0-28.5)	21.9 (15.8-28.0)	10.8 (8.0-13.6)	9.5 (6.9-12.2)
	Non-Hispanic	25.6 (22.0-29.3)	30.6 (26.1-35.1)	8.1 (7.2-8.9)	7.2 (6.4-8.0)
**Education**	No HS diploma	19.2 (14.4-24.0)	27.6 (21.2-34.0)	11.9 (9.7-14.1)	13.8 (11.2-16.4)
	HS diploma	23.8 (19.0-28.5)	26.7 (20.8-32.6)	8.5 (7.3-9.8)	7.9 (6.7-9.1)
	Some college	27.6 (22.7-32.5)	32.3 (26.6-38.0)	9.1 (7.5-10.6)	7.6 (6.3-8.9)
	Bachelor's or higher	35.5 (24.6-46.4)	35.9 (25.0-46.7)	5.5 (4.3-6.8)	3.6 (2.8-4.4)
**Income**	Less than $15k	23.4 (18.7-28.1)	28.0 (22.4-33.5)	15.0 (12.6-17.3)	14.4 (12.1-16.7)
	$15k - $25k	27.2 (21.4-33.0)	36.9 (28.6-45.3)	13.4 (11.2-15.6)	14.4 (12.3-16.4)
	$25k - $50k	30.1 (23.2-37.0)	35.4 (26.8-44.0)	7.6 (6.2-9.1)	6.9 (5.5-8.2)
	$50k +	22.0 (15.5-28.6)	18.1 (11.7-24.5)	4.4 (3.5-5.3)	2.5 (1.9-3.2)
**Marital Status**	Married	26.8 (21.9-31.7)	30.7 (25.2-36.2)	6.6 (5.7-7.6)	5.1 (4.4-5.9)
	Widowed	20.4 (12.8-28.0)	28.8 (18.8-38.9)	8.5 (7.3-9.8)	8.5 (7.3-9.7)
	Single, divorced, or separated	24.7 (20.7-28.7)	29.5 (24.5-34.4)	12.6 (10.5-14.7)	12.4 (10.5-14.3)
**Region**	Northeast	20.3 (13.1-27.4)	22.2 (16.7-27.8)	6.4 (4.5-8.3)	5.5 (3.5-7.6)
	Midwest	26.1 (17.5-34.7)	32.1 (22.0-42.1)	7.9 (5.7-10.0)	7.2 (4.9-9.4)
	West	26.1 (20.8-31.3)	33.7 (25.7-41.7)	9.5 (8.0-11.0)	9.6 (8.5-10.7)
	South	27.5 (20.4-34.7)	25.7 (18.2-33.1)	8.1 (6.4-9.8)	5.4 (3.7-7.1)
**Urban Density**	Metro	23.9 (20.0-27.9)	29.3 (24.1-34.5)	8.1 (7.1-9.1)	6.9 (6.0-7.9)
	Micro	28.4 (20.1-36.6)	32.2 (24.6-39.8)	9.1 (6.5-11.8)	9.4 (6.4-12.5)
	Rural	31.6 (18.9-44.2)	30.5 (22.0-39.0)	8.5 (5.4-11.5)	8.8 (7.1-10.6)
**General Health Status**	Excellent	9.8 (6.7-13.0)	17.0 (8.1-25.8)	5.1 (3.9-6.2)	2.7 (1.8-3.6)
	Very good	20.4 (15.9-25.0)	19.7 (15.4-24.1)	4.7 (3.8-5.5)	3.8 (3.0-4.7)
	Good	19.1 (13.6-24.6)	22.8 (17.7-27.9)	9.7 (8.4-11.0)	8.4 (7.4-9.4)
	Fair	30.9 (26.0-35.9)	34.2 (28.7-39.8)	14.9 (12.0-17.7)	16.0 (13.2-18.9)
	Poor	30.6 (24.8-36.4)	40.2 (30.8-49.6)	22.0 (16.4-27.6)	23.7 (17.2-30.3)
**No. Chronic Conditions**	Zero to 1	16.1 (12.2-20.0)	16.1 (11.3-21.0)	6.2 (4.9-7.5)	3.3 (2.4-4.2)
	2 to 3	23.2 (18.8-27.7)	30.4 (24.6-36.2)	7.6 (6.7-8.5)	5.8 (4.9-6.7)
	4 to 10	31.2 (25.3-37.2)	35.9 (29.4-42.3)	11.3 (9.6-12.9)	13.7 (12.3-15.1)
**Functional Limitations**	Zero ADLs	19.8 (15.5-24.0)	22.6 (18.4-26.8)	6.2 (5.3-7.0)	5.1 (4.3-5.8)
	1-2 ADLs	27.4 (21.6-33.2)	32.7 (25.9-39.6)	13.0 (10.7-15.2)	12.2 (10.3-14.1)
	3-6 ADLs	33.8 (26.9-40.7)	41.2 (32.4-50.0)	18.4 (14.9-21.9)	19.3 (16.6-22.0)
**Functional Limitations**	Zero IADLs	16.8 (12.5-21.1)	19.6 (15.0-24.2)	5.8 (5.1-6.6)	4.4 (3.7-5.1)
	1-2 IADLs	26.3 (21.1-31.4)	30.9 (25.0-36.8)	13.1 (11.1-15.1)	13.5 (11.7-15.4)
	3-6 IADLs	35.6 (28.4-42.7)	42.6 (33.1-52.1)	20.6 (16.5-24.8)	21.4 (17.3-25.5)
**Depression (PHQ)**	None or minimal	16.6 (12.6-20.5)	18.3 (14.2-22.4)	5.7 (4.9-6.4)	4.5 (4.0-5.1)
	Mild to severe	33.3 (28.3-38.3)	40.1 (33.5-46.6)	19.4 (16.8-22.0)	18.8 (16.1-21.5)
**Anxiety (GAD)**	No	21.2 (17.8-24.6)	23.7 (19.5-27.9)	6.7 (5.9-7.5)	5.4 (4.8-6.1)
	Yes	37.2 (31.3-43.1)	46.3 (38.4-54.2)	23.8 (20.2-27.4)	24.9 (21.7-28.1)
**Supplemental Insurance**	Medicaid	28.7 (21.6-35.8)	35.1 (26.3-43.8)	14.2 (11.2-17.1)	12.5 (10.3-14.7)
	Medicare Advantage	18.8 (11.9-25.6)	22.1 (14.7-29.5)	4.2 (3.2-5.2)	3.3 (2.6-4.1)
	FFS with employer/retiree	18.5 (11.9-25.0)	29.7 (18.8-40.7)	6.1 (4.7-7.4)	4.3 (3.2-5.3)
	FFS with self-purchased or other	37.1 (29.7-44.4)	37.4 (29.2-45.5)	9.0 (7.7-10.2)	8.4 (7.0-9.7)
	FFS with no supplement	21.6 (17.3-26.0)	26.8 (21.3-32.3)	15.7 (12.9-18.5)	16.6 (13.3-19.8)
	**TOTAL POPULATION**	**25.2 (21.8-28.6)**	**29.8 (25.6-34.1)**	**8.3 (7.4-9.1)**	**7.4 (6.6-8.2)**

eTable 1. Detailed prevalence analyses of delaying care due to cost and having problems paying medical bills, by demographic and health characteristics

© 2021 Madden JM et al. *JAMA Health Forum.*

eTable 2. Demographic and health characteristics of the study population by supplemental insurance type

	Supplemental Insurance Type:	Enrollees under 65 y					Enrollees 65 and over				
		Medicaid	Medicare Advantage	FFS with employer/ retiree	FFS with self-purchased or other	FFS with no supplement	Medicaid	Medicare Advantage	FFS with employer/ retiree	FFS with self-purchased or other	FFS with no supplement
Characteristic		%	%	%	%	%	%	%	%	%	%
Age group	18-54 y	53.7	30.0	37.6	15.2	42.7					
	55-64 y	46.3	70.0	62.4	84.8	57.3					
	65-74 y						53.6	54.6	61.0	55.0	61.2
	75-84 y						32.2	33.3	27.9	32.8	28.0
	85 y +						14.2	12.1	11.1	12.2	10.8
Sex	Male	50.4	51.5	49.8	53.2	60.8	30.2	44.4	48.7	43.3	52.7
	Female	49.6	48.5	50.2	46.8	39.2	69.8	55.6	51.3	56.7	47.3
Race	Afro Am	23.2	15.6	10.2	15.3	16.4	22.2	8.8	7.2	2.4	10.2
	White	66.5	72.8	77.7	77.9	68.1	57.6	83.0	84.5	92.7	77.3
	Other	7.2	10.3	9.8	4.9	12.2	13.1	5.9	6.5	4.0	9.9
Ethnicity	Hispanic	12.8	9.2	4.2	5.5	6.6	24.7	8.9	5.1	2.2	6.2
	Non-Hispanic	86.5	90.2	95.8	94.5	92.7	75.0	90.6	94.3	97.4	93.3
Education	No HS diploma	31.3	11.4	5.1	7.9	20.3	45.4	14.6	5.9	8.6	18.6
	HS diploma	33.7	30.5	30.0	24.9	37.2	26.6	26.6	20.7	25.6	28.7
	Some college	29.3	42.9	47.8	51.1	30.4	19.1	30.9	31.4	31.5	30.9
	Bachelor's or higher	4.6	15.0	16.0	16.1	12.1	8.3	27.6	41.6	34.1	21.8
Income	Less than $15k	75.1	22.6	5.2	14.5	36.3	74.3	9.0	3.9	6.7	19.7
	$15k - $25k	18.5	31.2	16.6	24.5	24.8	18.7	18.7	9.0	15.4	22.9
	$25k - $50k	6.2	29.3	25.5	35.6	23.3	4.8	32.9	26.7	29.7	31.6
	$50k +	0.2	16.9	52.7	25.4	15.7	2.2	39.4	60.3	48.1	25.8
Marital Status	Married	16.3	46.7	72.8	42.1	35.2	22.3	58.4	67.0	59.6	47.0
	Widowed	7.4	6.8	2.4	11.8	4.6	33.7	23.6	19.7	24.0	26.2
	Single, divorced, or separated	76.1	46.5	24.8	46.2	60.3	43.8	17.9	13.2	16.3	26.7
Region	Northeast	16.8	13.6	27.6	32.1	20.5	18.5	17.4	21.3	17.1	16.9
	Midwest	18.7	24.1	19.8	23.6	20.6	15.7	23.8	17.9	27.3	23.6
	South	46.8	44.2	36.9	28.5	44.2	43.1	33.9	37.8	38.1	39.6
	West	17.7	18.1	15.7	15.8	14.7	22.7	24.8	23.0	17.6	19.9
Urban Density	Metro	76.5	80.8	79.3	85.1	70.2	79.2	87.5	81.7	73.5	73.3
	Micro	15.1	11.6	12.7	10.2	17.9	11.7	7.2	13.1	17.1	16.0
	Rural	8.4	7.5	8.0	4.7	11.9	9.1	5.2	5.2	9.4	10.6
General Health Status	Excellent	5.4	2.7	3.2	2.0	3.7	9.0	20.4	20.2	20.6	19.3
	Very good	9.5	8.6	8.0	13.7	6.5	18.4	34.1	38.7	34.9	30.9
	Good	30.0	32.1	33.8	34.8	32.7	33.5	30.9	29.3	30.7	28.9
	Fair	34.2	37.8	33.5	29.1	30.9	28.4	11.3	9.6	10.5	14.9
	Poor	20.5	18.2	19.9	20.3	25.1	10.5	2.9	2.1	3.2	4.8
No. Chronic Conditions	Zero to 1	22.6	12.7	19.9	17.1	20.3	16.7	26.9	27.7	26.3	31.2
	2 to 3	36.7	39.4	38.8	45.5	38.4	37.1	47.4	46.8	47.0	41.1
	4 to 10	39.6	46.7	40.6	37.3	38.8	45.0	24.9	25.0	25.7	26.2
Functional Limitations	Zero ADLs	48.5	46.4	49.0	55.5	45.3	53.8	77.3	79.9	78.8	70.5
	1-2 ADLs	26.4	32.6	28.2	25.3	33.4	25.6	16.3	14.1	15.2	20.7
	3-6 ADLs	25.1	21.0	22.8	19.2	21.3	20.5	6.5	5.9	6.0	8.5
Functional Limitations	Zero IADLs	34.9	29.9	36.6	44.2	35.7	52.1	75.2	77.4	73.9	73.0
	1-2 IADLs	38.4	44.1	38.5	37.2	37.3	29.3	19.6	18.2	20.8	19.8
	3-6 IADLs	25.8	26.0	24.9	18.0	26.9	18.0	5.1	4.3	5.2	6.8
Depression (PHQ)	None or minimal	32.7	34.8	34.3	40.4	32.0	52.2	78.0	80.2	77.9	69.8
	Mild to severe	53.0	59.9	58.6	51.8	54.6	31.1	17.3	14.8	16.7	21.8
Anxiety (GAD)	No	52.0	59.2	61.5	80.9	55.5	64.7	87.0	89.0	87.3	78.1
	Yes	33.6	35.5	31.5	11.4	31.2	18.5	8.3	6.0	7.3	13.5

© 2021 Madden JM et al. *JAMA Health Forum.*

eTable 3. Sensitivity Analysis – Multivariate modeling results without supplemental insurance type

		Enrollees under 65 y		Enrollees 65 y and over	
		Delayed Care Due to Cost	Problems Paying Medical Bills	Delayed Care Due to Cost	Problems Paying Medical Bills
**Characteristic**	***Reference category***	O.R. (95% C.I)	O.R. (95% C.I)	O.R. (95% C.I)	O.R. (95% C.I)
Aged 55-64 y	*18-54*	0.91 (0.66-1.24)	1.15 (0.84-1.56)		
Aged 75-84 y	*65-74 y*			**0.53 (0.44-0.63)**	**0.60 (0.46-0.79)**
Aged 85+ y				**0.26 (0.19-0.36)**	**0.32 (0.23-0.44)**
Female	*Male*	1.22 (0.93-1.60)	1.05 (0.77-1.42)	1.10 (0.90-1.35)	0.97 (0.78-1.20)
Black race	*White*	0.81 (0.53-1.26)	1.14 (0.79-1.64)	1.11 (0.81-1.52)	**2.14 (1.61-2.83)**
Other race		0.73 (0.40-1.32)	0.77 (0.44-1.33)	1.34 (0.91-1.96)	1.08 (0.74-1.56)
Hispanic ethnicity	*Non-Hispanic*	0.90 (0.46-1.75)	0.68 (0.39-1.21)	0.84 (0.63-1.13)	0.90 (0.62-1.32)
No high school diploma	*Bachelor's or higher*	**0.49 (0.30-0.79)**	0.59 (0.33-1.05)	1.01 (0.69-1.49)	**1.47 (1.06-2.03)**
High school diploma		0.68 (0.43-1.09)	0.71 (0.41-1.20)	0.98 (0.71-1.35)	1.18 (0.89-1.57)
Some college		0.70 (0.43-1.15)	0.82 (0.50-1.35)	1.08 (0.81-1.45)	1.28 (0.98-1.68)
Income < $15k	*$50K+*	1.23 (0.72-2.08)	**2.15 (1.08-4.29)**	**2.77 (1.92-4.01)**	**2.75 (1.91-3.98)**
Income $15k - $25k		1.41 (0.84-2.34)	**2.86 (1.48-5.55)**	**2.86 (2.11-3.88)**	**3.83 (2.81-5.21)**
Income $25k - $50k		1.54 (0.89-2.67)	**2.44 (1.26-4.69)**	**1.65 (1.23-2.21)**	**2.09 (1.44-3.05)**
Widowed	*Married*	0.77 (0.41-1.43)	0.85 (0.42-1.75)	0.98 (0.76-1.26)	1.11 (0.84-1.47)
Single, divorced, or separated		1.10 (0.79-1.54)	1.03 (0.72-1.47)	1.12 (0.86-1.44)	**1.36 (1.06-1.73)**
Midwest region	*Northeast*	1.18 (0.65-2.13)	1.47 (0.98-2.23)	1.20 (0.87-1.65)	1.31 (0.85-2.03)
West region		1.46 (0.86-2.50)	1.29 (0.78-2.14)	**1.31 (1.02-1.68)**	1.11 (0.74-1.68)
South region		1.31 (0.81-2.12)	**1.81 (1.24-2.64)**	1.25 (0.99-1.57)	1.41 (0.99-2.01)
Micro	*Metro*	1.31 (0.90-1.91)	1.20 (0.83-1.73)	1.03 (0.81-1.31)	**1.33 (1.03-1.72)**
Rural		1.48 (0.82-2.65)	0.94 (0.59-1.49)	0.81 (0.61-1.07)	0.94 (0.64-1.37)
Fair or poor health	*Excell., v. good, good*	1.19 (0.85-1.66)	1.11 (0.77-1.60)	1.23 (0.94-1.60)	1.22 (0.96-1.56)
2-3 chronic conditions	*0-1*	1.18 (0.74-1.88)	**1.90 (1.13-3.18)**	1.01 (0.77-1.33)	**1.49 (1.10-2.01)**
4-10 chronic conditions		1.42 (0.82-2.43)	**1.79 (1.06-3.02)**	0.85 (0.65-1.13)	**2.03 (1.51-2.72)**
1-2 Limitations in ADLs	*None*	1.06 (0.69-1.61)	1.08 (0.78-1.49)	**1.41 (1.08-1.84)**	1.07 (0.81-1.42)
3-6 Limitations in ADLs		1.15 (0.74-1.80)	1.29 (0.87-1.92)	**1.43 (1.06-1.94)**	1.19 (0.85-1.66)
1-2 Limitations in IADLs	*None*	1.30 (0.88-1.93)	1.30 (0.89-1.92)	**1.66 (1.36-2.03)**	**2.25 (1.70-2.97)**
3-6 Limitations in IADLs		**1.76 (1.05-2.96)**	**2.00 (1.30-3.09)**	**2.22 (1.58-3.12)**	**2.66 (1.76-4.01)**
Depression sx: mild to severe	*None or minimal*	**1.51 (1.03-2.22)**	**1.68 (1.16-2.43)**	**1.92 (1.47-2.51)**	**1.65 (1.23-2.22)**
Anxiety sx, yes	*No*	**1.47 (1.08-1.99)**	**1.81 (1.27-2.58)**	**1.69 (1.22-2.34)**	**2.07 (1.59-2.70)**

© 2021 Madden JM et al. *JAMA Health Forum.*

eFigure. Extension analysis examining association between lower income status and unaffordability by supplemental insurance type: Estimated odds ratios comparing risk of unaffordability associated with lower incomes (as indicated) compared with income $50K+, by coverage type

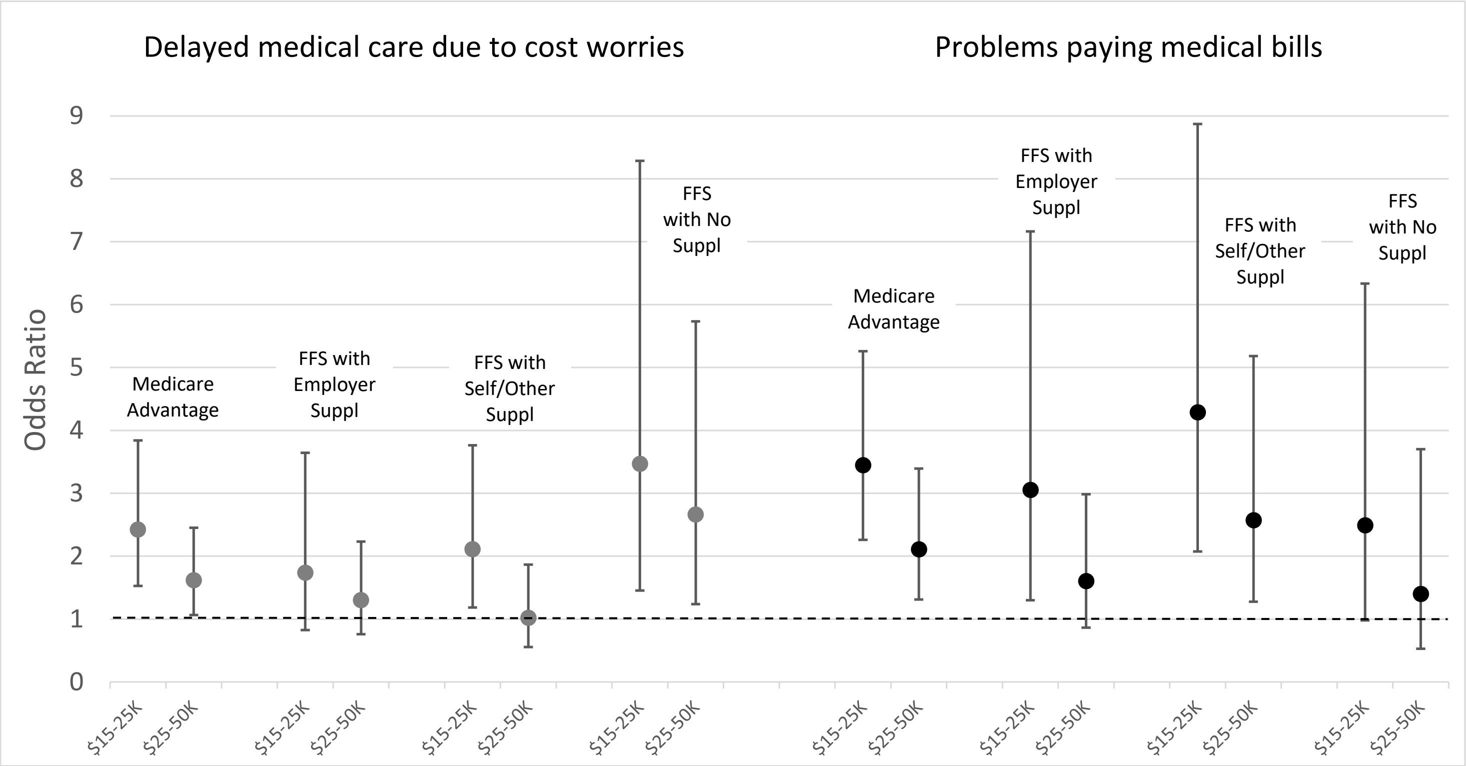

Details on methods and findings: For each outcome (delayed care and problems paying), we constructed 4 logistic regression models based on the main models in Table 3 of the article. These extension models included the population aged 65 and over only and excluded individuals in the lowest income category (<$15K) and those with any Medicaid coverage. Model Ns were 8503 and 8500 for each outcome, respectively. Income $50K or higher was the income reference category in all cases. The reference category for supplemental insurance type varied among models: Medicare Advantage; FFS with employer/retiree; FFS with self-purchased or other; and FFS with no supplement. All models included terms for interactions between income level and supplemental insurance type. Estimated odds ratios (ORs, in Figure above and left column of Table below) compare the odds of unaffordability in the income categories indicated, compared to those with incomes > $50k. "Ratios of odds ratios" (ORRs) in the Table below test for differences in OR results among the insurance types. We detected no statistically significant differences in the ORs for lower vs higher income among insurance types, except for: delayed care, self-purchased vs no supplement, income $25-50K vs>$50K. All models controlled for age group, sex, race, ethnicity, education, marital status, census region, urban/rural status, general health status, number of chronic conditions, ADLs, IADLs, and depression and anxiety symptoms. Limitations include moderate sample size and unobserved variables.

© 2021 Madden JM et al. *JAMA Health Forum.*

eTable 4. Extension Analysis Examining Association Between Lower Income Status and Unaffordability by Supplemental Insurance Type: Multivariate modeling results with interaction of income level and supplemental insurance type, varying the insurance reference category

Unaffordability outcome / Supplemental insurance type / Income category	Reference group	Increased odds of unaffordability associated with having lower income		RATIO of ORs -- to determine whether the elevated risk associated with having lower income is significantly greater under some forms of insurance vs others					
				vs FFS w ESI		vs FFS with self/other		vs FFS w no supp	
		OR	95% CI	ORR	95% CI	ORR	95% CI	ORR	95% CI
**Delayed medical care due to cost worries**									
Given Medicare Advantage									
Income $15k - $25k	versus $50K+	**2.42**	**1.53 - 3.84**	1.40	0.63 - 3.10	1.15	0.58 - 2.28	0.70	0.28 - 1.73
Income $25k - $50k	versus $50K+	**1.62**	**1.07 - 2.45**	1.24	0.64 - 2.39	1.58	0.79 - 3.18	0.61	0.27 - 1.37
Given FFS with employer/retiree									
Income $15k - $25k	versus $50K+	1.74	0.83 - 3.64			0.82	0.32 - 2.14	0.50	0.15 - 1.70
Income $25k - $50k	versus $50K+	1.30	0.76 - 2.23			1.28	0.59 - 2.77	0.49	0.19 - 1.25
Given FFS with self-purchased or other									
Income $15k - $25k	versus $50K+	**2.11**	**1.18 - 3.76**					0.61	0.22 - 1.71
Income $25k - $50k	versus $50K+	1.02	0.56 - 1.87					**0.38**	**0.16 - 0.93**
Given FFS with no supplement									
Income $15k - $25k	versus $50K+	**3.47**	**1.45 - 8.29**						
Income $25k - $50k	versus $50K+	**2.66**	**1.24 - 5.73**						
**Problems paying medical bills**									
Given Medicare Advantage									
Income $15k - $25k	versus $50K+	**3.45**	**2.26 - 5.26**	1.13	0.43 - 2.98	0.80	0.32 - 2.01	1.38	0.50 - 3.81
Income $25k - $50k	versus $50K+	**2.11**	**1.31 - 3.39**	1.31	0.62 - 2.79	0.82	0.37 - 1.80	1.51	0.57 - 4.02
Given FFS with employer/retiree									
Income $15k - $25k	versus $50K+	**3.05**	**1.30 - 7.17**			0.71	0.26 - 1.96	1.23	0.39 - 3.88
Income $25k - $50k	versus $50K+	1.61	0.86 - 2.99			0.62	0.23 - 1.68	1.15	0.39 - 3.40
Given FFS with self-purchased or other									
Income $15k - $25k	versus $50K+	**4.29**	**2.07 - 8.87**					1.72	0.53 - 5.58
Income $25k - $50k	versus $50K+	**2.57**	**1.28 - 5.18**					1.84	0.60 - 5.62
Given FFS with no supplement									
Income $15k - $25k	versus $50K+	2.49	0.98 - 6.34						
Income $25k - $50k	versus $50K+	1.40	0.53 - 3.70						

© 2021 Madden JM et al. *JAMA Health Forum.*
